# Establishment of a no-notice drill mode evaluation system for public health emergencies

**DOI:** 10.1371/journal.pone.0266093

**Published:** 2022-04-04

**Authors:** Sicheng Huang, Zibo Lin, Xinqi Lin, Lin Li, Feng Ruan, Wenhua Mei, Sidong Chen

**Affiliations:** 1 Zhuhai Center for Disease Control and Prevention, Zhuhai, Guangdong, China; 2 Guangdong Pharmaceutical University, Guangzhou, Guangdong, China; University of Defence in Belgrade, SERBIA

## Abstract

**Objective:**

At present, there are some no-notice drill mode evaluation systems for public health emergencies in Chinese hospitals, which are the subjects of assessment in this study. However, there is a lack of CDC. This study builds a set of no-notice drill mode evaluation systems for public health emergencies that involve the CDC.

**Methods:**

The indexes for these systems were based on the performance of two no-notice drills for public health emergencies in Guangdong Province. Twenty experts were invited to screen the indicators during two rounds of the Delphi method to determine the weight of first- and second-level indexes through the analytic hierarchy process, and the weight of the third-level index was calculated using the percentage method.

**Results:**

After two rounds of expert consultation, we obtained four first-level indicators, twenty-six second-level indicators and eighty-six third-level indicators. According to the weight calculated by analytic hierarchy process, the weights of the first-level indicators are emergency preparation (0.2775), verification and consultation regarding an epidemic situation (0.165), field investigation and control (0.3925) and summary report (0.165). Sensitivity analysis shows that the stability of the index is good.

**Conclusion:**

The no-notice drill mode evaluation system for public health emergencies constructed in this study can be applied to public health departments such as the CDC. Through promotion, it can provide a scientific basis for epidemiological investigation assessment.

## Introduction

In recent years, the new coronavirus pneumonia pandemic has swept the world. To test and evaluate the capacity of health emergency teams to respond to public health emergencies, many provinces and cities in China have strengthened emergency drills. The drill for public health emergencies can clarify the responsibilities and tasks of personnel at all levels, train the health emergency team, and take correct actions in the response process. At present, most emergency drills in China are conducted according to a drill script, which is helpful in familiarizing individuals with the process and improving emergency preparedness.

However, due to the particularity and complexity of public health emergencies, the key to managing such a public health crisis is epidemiological investigation and control of disease spread. If we make mistakes in judgment, it may lead to an epidemic or pandemic. Therefore, in emergency drills, we should focus on strengthening epidemiological investigations and the ability to control disease spread. The no-notice drill is exactly the direction these drills should take. The party participating in the exercise should not know the epidemic scenario in advance. Participants in the exercise can only assess the situation after completing the investigation on site. This achieves the purpose of assessment. To construct the no-notice drill mode evaluation system for public health emergencies, the Delphi method and analytic hierarchy process (AHP) were used in this study.

### Problem description

In the available literature, research on no-notice drills for public health emergencies at home and abroad is relatively limited: most studies focus on emergency disposal and emergency treatment of mass casualties [1–3], mass evacuation [4] and no-notice drill of mass vaccination [5]. In addition, there are studies on no-notice drills for public health emergencies, such as the Ebola no-notice drill held in Taiwan in 2014 [6] and a no-notice drill held in New York City for respiratory infectious diseases such as measles and influenza in 2015 [7]. However, these exercises mainly evaluated the hospital’s emergency preparedness for public health emergencies and failed to assess the ability of epidemiological investigators in an epidemic situation4. In addition, researchers have not thoroughly studied the evaluation system for the no-notice drill.

In addition, the existing research related to the no-notice drill for public health emergencies is mainly limited to the implementation of the drill, the evaluation process and result analysis, and there is a lack of the construction process of the evaluation system. The evaluation system of the no-notice drill has not been deeply studied. The construction of these drill evaluation systems mainly relies on some existing drill guidelines, such as the “Hospital Surge Evaluation Tool” used to evaluate the emergency response capacity of mass casualties [3], the Homeland Security Exercise and Evaluation Program (HSEEP) [5, 7], the hospital evaluation standards and hospital infection control guidelines issued by China [6], and some of them adopt the simple Delphi method [4]. Compared with the Delphi-AHP, these studies are arbitrary and lack scientificity in the selection of indicators, especially in the determination of weight, which is not conducive to the evaluation of emergency capacity.

To solve the two key problems mentioned above, improve the epidemiological investigation ability of personnel in public health departments, and develop scientific evaluation tools, this study improves the existing no-notice drill mode evaluation system for public health emergencies and develops a set of evaluation tools suitable for flow investigators. The indexes are screened, and the weight is determined by the Delphi method and AHP.

The Delphi method is an effective group consensus consultation method that is widely used in the fields of medicine and public health [8–10]. It includes a literature review, stakeholder ideas and expert judgment. The research results are designed and collected by an anonymous expert consultation questionnaire [11–13], which has high reliability. Because the Delphi method is mainly aimed at qualitative research [14, 15], it is often combined with the AHP in qualitative and quantitative research [16–19].

The AHP was proposed by Thomas Saaty (1980). To date, this method has undergone many modifications. In recent research, to overcome some defects of this method, the AHP has been combined with fuzzy logic theory [20, 21], which models basic information and approximations [20–22]. In addition, in view of the excessive number of pairwise comparisons, many experts have also made modifications on the basis of the AHP [23, 24] and formulated the BWM, FUCOM [25] and other methods, especially the BWM, which has been applied often in recent years [26, 27]. However, in some cases, the AHP is still used in its original form [28, 29]. The AHP is widely used in the construction of evaluation systems [30–32]. By comparing the opinions of experts, the quantitative relationship between the elements of the same level and the elements of the upper level is determined to assign the relative important weight of the lowest level (schemes and measures for decision-making) relative to the highest level (overall goal).

## Describe the application method

### Delphi method

**Fig 1:**
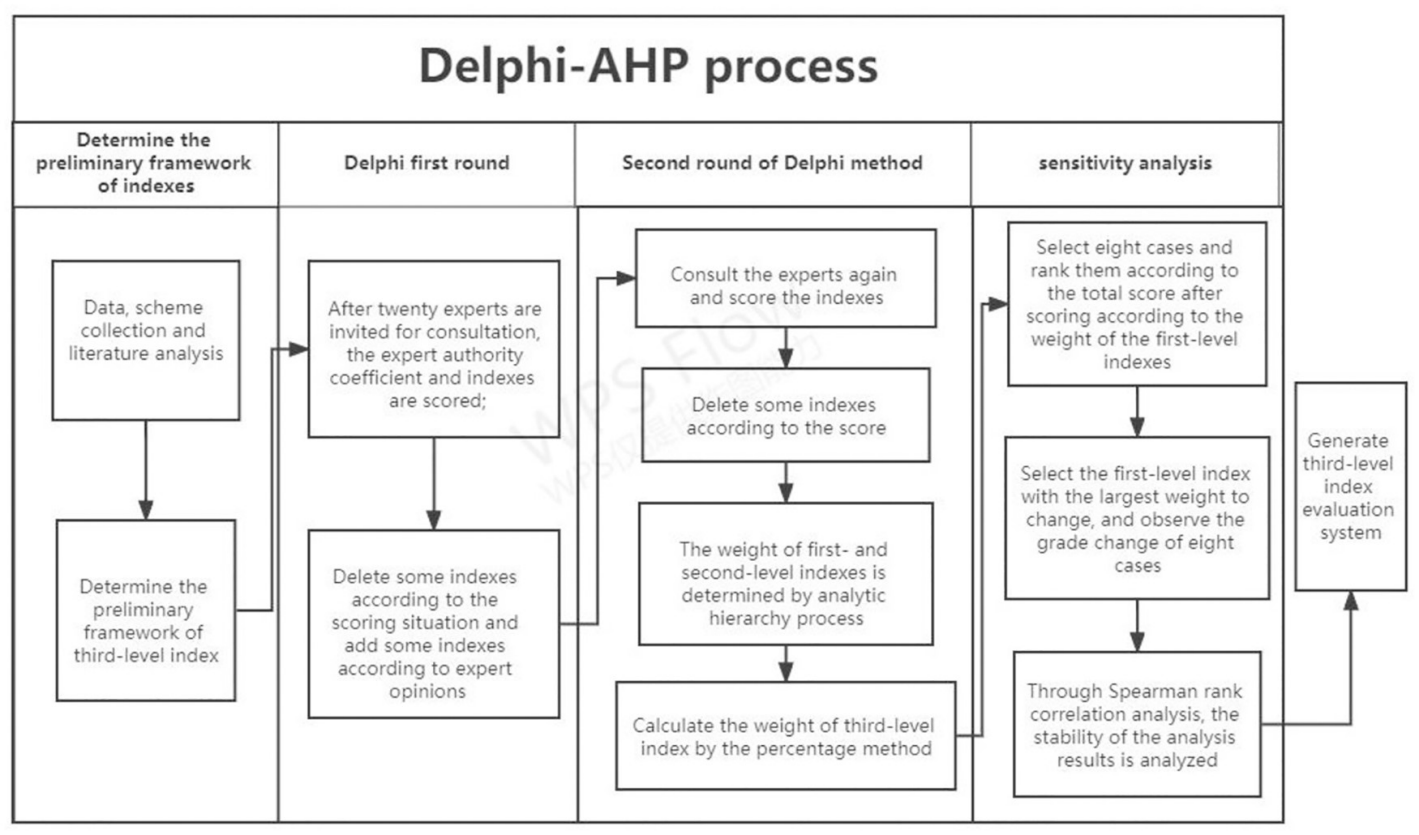
Delphi-AHP process.

The Delphi method can be applied to the establishment of various evaluation index systems and the determination process of specific indicators. Through several rounds of feedback, we made full use of and absorbed the experience and knowledge of experts so that the opinions of the experts gradually converged. In this study, we planned to invite approximately twenty domestic experts in the field of public health to screen and revise the indicators through two rounds of the Delphi method to determine the indicators and weights (see Fig 1).

The first round constructed evaluation indicators. We considered the basic steps of infectious disease outbreak investigation [33, 34], the guide to health emergency drills prepared by the central disease control, the emergency plan and technical scheme for the emergency disposal of public health emergencies in Guangdong Province, and the framework of the simple scoring table of the two no-notice drills held in China in 2015 and 2016 (formulated under the guidance of the emergency management experts of Guangzhou Center for disease prevention and control) [35]. This round was modified and developed into a preliminary framework of evaluation indicators.

Before issuing the questionnaire, we invited twenty-six domestic public health experts to participate in our study, and twenty agreed to participate. Participants were asked to form an expert group via text messages and e-mail, and the group included university professors from Southern Medical University and the School of Public Health of Sun Yat sen University, experts and managers who have long been engaged in front-line treatment of infectious diseases from Guangdong Provincial Health Commission, Guangdong Emergency Hospital, Guangdong Provincial and Municipal CDC. Among them, sixteen had participated in at least one no-notice drill and were responsible for the participants, evaluation team and expert group in the drill. Therefore, they have a certain understanding of this drill mode.

There are two rounds of Delphi consultation. In the first round, the expertise of participants and their familiarity with the disease scenario are evaluated, giving us the expert authority coefficient (Cr). At the same time, experts score the importance of the first- and second-level indexes [15]. The score is divided into five levels from high to low according to a Likert scale (5 points are very important and 1 point is very unimportant). Considering that it is difficult to carry out and assess the no-notice drill, the third level index not only evaluates the importance score but also scores the feasibility. The scoring standard is also divided into five levels from high to low according to the Likert scale. After collecting data from the first round, the coefficient of variation CV of the third-level indicators were calculated, and the indicators that cannot meet the importance (or feasibility) 16 assignment mean≥3.65 and the coefficient of variation < 0.25 were eliminated. At the same time, open suggestions were taken, and suggestions mentioned by at least two experts were selected as new indicators.

In the second round, the revised indicators were distributed. The distribution object was the experts who provided feedback in the first round, and the evaluation content included the importance scores, the feasibility score and the variation coefficient of the third-level indicators. The indexes that cannot meet the mean value of importance and feasibility assignment ≥3.65 and the coefficient of variation < 0.25 were eliminated. The average value of the importance assignment of the first- and second-level index was transformed into a judgment matrix, and the weight of each first- and second-level index was calculated by the AHP.

This study uses an AHP to determine the weight of evaluation indicators of health emergency drills, which mainly follows these steps:

1. **Establish hierarchical model**
  This is generally divided into two layers: the top layer is the target layer, and the bottom layer is the standard layer.
2. **Construct judgment matrix**
  The values of judgment matrix elements reflect people’s understanding of the relative importance of various factors. Generally, the judgment matrix is constructed by pairwise comparison between indicators, specifically using the 1~9 scale of scholar GWM van der Staay (see Table 1).

**Table 1:** Scale of relative importance.

Scale	meaning	explain
1	Equally important	Both contribute equally to the goal
3	Slightly important	One is slightly better than the other
5	Obviously important	One evaluation is more favorable than the other
7	Very important	One evaluation is more favorable than the other, and its advantages have been proven in practice
9	Absolutely important	The degree of apparent importance can be asserted as the highest
2,4,6,8	The intermediate value of the above two adjacent judgments	When a compromise is required
The reciprocal of the above nonzero values	If one of the above nonzero numbers is specified when comparing index I with index J, index J and index I have the reciprocal of the corresponding nonzero number	Indicates different degrees of "unimportant"

According to the Staay scale, the judgment matrix is constructed. The first-level index and the second-level index were compared to calculate the weight Wi of each index.

(1) First, the eigenvector of the judgment matrix is determined, which is also the relative weight of each factor.


$$Set\;comparison\;judgment\;matrix\;A=\left[\begin{array}{cccc} a_{11} & a_{12} & \ldots & a_{1\text{n}}\\ a_{21} & a_{22} & \ldots & a_{2\text{n}}\\ \ldots & \ldots & \ldots & \ldots \\ a_{\text{n}1} & a_{\text{n}2} & \ldots & a_{\text{nn}} \end{array}\right]$$

#### Sum product method

A, Normalize each column of judgment matrix A $b_{\text{ij}}=\frac{a_{\text{ij}}}{\sum_{i=1}^{n}a_{\text{ij}}}$ (i, j = 1,2,,, n)

B, The normalized judgment matrix of each column is added by row $\bar{W}=\sum_{j=1}^{n}b_{\text{ij}}$ (i, j = 1,2,,, n)

C, Normalize vector $\bar{W}={\left[\begin{array}{cccc} \bar{W_{1}} & \bar{W_{2}} & \ldots & \bar{W_{\text{n}}} \end{array}\;\right]}^{T}:\;W_{i}=\frac{\bar{{\text{W}}_{\text{i}}}}{\sum_{\text{i}=1}^{\text{n}}\bar{{\text{W}}_{\text{i}}}}$ (i = 1, 2,,, n) Then $\bar{W}={\left[\begin{array}{cccc} \bar{W_{1}} & \bar{W_{2}} & \ldots & \bar{W_{\text{n}}} \end{array}\;\right]}^{T}$ is the calculated feature vector, that is, the weight of each second-level index.

Finally, the consistency of the indicators is tested. First, calculate the maximum eigenvalue of the judgment matrix: $\lambda_{\text{max}}=\sum_{i=1}^{n}\frac{{\left(\text{AW}\right)}_{\text{i}}}{nW_{\text{i}}}$. Recalculate the consistency index: $CI=\frac{\lambda_{\text{max}}-n}{n-1}$. The random consistency index RI can be obtained by looking up the table. Consistency test: $CR=\frac{CI}{CR}$. If the consistency ratio of the discriminant matrix CR<0. 1, the consistency of the judgment matrix is qualified [16].

After the importance of the third-level indicators is assigned and the corresponding score is calculated by the percentage assignment method, the final weight of each third-level indicator is calculated by the percentage method [15].

The index system validity evaluation evaluates content validity and structure validity. Content validity mainly depends on the correctness of the whole research method and step calculation process. In this study, on the basis of reviewing the literature, we formulated the evaluation framework and index content selection criteria. Then, two rounds of Delphi expert consultation were conducted to select and modify indicators. The evaluation index includes the basic content to be evaluated.

The indicator system reliability evaluation uses Cronbach’s alpha coefficient to evaluate the internal reliability of the index system, and α>0.80 was the criterion for determining the reliability of the index. Finally, sensitivity analysis was carried out by changing the weight coefficient of the criterion.

## Results

### Basic information

In this study, all experts had high academic achievements in their respective fields. Nineteen (95%) were provincial and municipal experts, 19 (95%) had graduate-level educations or more, and 19 (95%) had senior deputy titles or above. They are front-line personnel or emergency management experts who had been engaged in public health work for an average of 23.9 (12–44) years (see Table 2). This round demonstrated that the basic advice from experts was helpful. In this study, twenty questionnaires were distributed in two rounds of the Delphi survey, and nineteen were recovered, for an effective recovery rate of 95%. The questionnaire recovery rate was high. The positive coefficient of the two rounds of experts was 95%. The expert authority coefficient (CR) was 0.805 (> 0.8), indicating that the expert consultation results were accurate and reliable [36].

**Table 2:** Demographics of the Delphi survey experts.

	participant	quantity (n)	percentage(%)
Gender	male	16	80
	female	4	20
Age (years)	36–45	9	45
	46–55	10	50
	56–65	1	5
Education	undergraduate	9	45
	master	10	50
	doctor	1	5
Professional title	positive advanced	13	65
	deputy senior	6	30
	intermediate	0	0
	primary	1	5
Professional field	public health	15	75
	health management	5	25
	clinical medical treatment	0	0
	other	0	0
Nature of work	administrative management	6	20
	business technology	14	80
	other	0	0
Familiarity	very familiar	3	15
	familiar	13	65
	quite familiar	4	20
	unfamiliar	0	0
Working time (year)	1–10	0	0
	11–20	7	35
	21–30	8	40
	more than 30	5	25

#### Concentration of expert opinions

In this study, the concentration and coordination of expert opinions (average score and coefficient of variation CV of third-level indicators) of each index were calculated. After two rounds of expert consultation, the average comprehensive score of third-level indicators increased from 4.29 (3.99 to 4.74) to 4.56 (4.25 to 4.80), and the concentration of expert opinions increased significantly.

#### Index screening results

In the first round of the survey, the average CV was 0.19 (0.10 to 0.31), and four third-level indicators were greater than 0.25. According to the scores and opinions of experts, one second-level indicator and seven third-level indicators were removed, and one second-level indicator and four third-level indicators were added. In the second round of the survey, the average coefficient of variation was 0.10 (0.06 to 0.21), and no index was greater than 0.25, indicating that the opinions of experts tended to be consistent [37].

Based on two rounds of expert opinions, a no-notice drill evaluation index system composed of four first-level indicators, twenty-six second-level indicators and eighty-six third-level indicators was finally formed. The main indicators were as follows: (1) emergency preparedness: preparation of personnel, materials and plans; information transmission and response speed; (2) epidemic situation verification: verification and preliminary investigation of the incident; and (3) field investigation and control: case epidemiological investigation, external environment sampling, preliminary report and information release; (4) Summary report: whether the content of the investigation report is comprehensive and whether there is discussion, summary and reflection.

#### Weight of indicators

The AHP and percentage method were used to determine the weights of various indicators of the no-notice drill evaluation system for public health emergencies, as shown in Table 3. The weights of the four first-level indexes were emergency preparation (0.2775), verification and consultation regarding an epidemic situation (0.165), field investigation and control (0.3925) and summary report (0.165). Among them, the field investigation and control subindexes were ranked the highest, and their weight was the heaviest.

**Table 3:** Evaluation index and weight of no-notice drills for public health emergencies.

First-level index weight	Second-level index	Third-level index	Weight
Emergency preparedness 0.2775	Information transfer 0.0943	Report and register after receiving the report	0.0233
		After receiving the report, the emergency information is transmitted and shared within the department	0.0239
		Report to the leadership of the unit in time	0.0231
		A unit leader carries out instructions	0.0240
	Start up a plan 0.0135	When necessary, experts should be convened to conduct risk assessment on the nature, type and degree of damage.	0.0018
		Expert group personnel choose whether to match event characteristics	0.0017
		Whether effective information can be provided for expert group assessment	0.0017
		Whether the categories, warning levels, and response levels are correctly determined	0.0017
		Whether a response is put forward	0.0016
		According to the plan, start the corresponding emergency response level	0.0016
		Rapid dispatch of various units and emergency linkage departments	0.0017
		According to the nature of the incident, it is necessary to make recommendations to the health administration department when contingency plans are necessary	0.0017
	Team formation 0.051	Setting up an emergency coordination leadership group	0.0127
		The composition of emergency team personnel can meet the needs of event handling	0.0132
		The proportion of the staff of each professional group is appropriate	0.0130
		The emergency team’s technical level and management experience are properly distributed	0.0120
	Emergency material preparation 0.0642	Communication equipment	0.0203
		Life guarantee	0.0226
		Professional materials	0.0212
	Preparation of knowledge documents 0.0208	Supporting technical documents and various work forms	0.0208
	Time limit for action 0.0337	The emergency team arrived at the scene within the specified time	0.0337
Verification and consultation of the epidemic 0.1650	Event verification 0.0443	Before departure, we should know the general situation of the incident	0.0443
	When you arrive at the scene, contact the local personnel to understand the situation 0.0693	Understand the local basic situation	0.0228
		Understand the development process of events	0.0233
		The management of local events	0.0232
	Outbreaks in the field 0.0513	The members of the conference include the heads of the teams, the team leaders, and the people on the scene	0.0065
		A preliminary descriptive analysis of the nature of the epidemic and the incident was made	0.0064
		Definition of the case of case and the definition of close contacts	0.0066
		Whether the case search method and scope are established	0.0065
		Whether the search method and scope of the case are reasonable	0.0064
		Whether or not a quality control indicator is proposed for case search	0.0061
		A preliminary judgment of the nature of the incident and report to the higher authorities	0.0064
		Formulate an investigation plan	0.0065
Field investigation and control 0.3925	Whether the field command has a reasonable division of labor in the field 0.0427	Send a survey of professionals to each scene	0.0221
		The team consists of an investigation group, a sterilization group, an inspection group, a health education group, a logistic support group and a medical group (the teams are grouped according to the situation)	0.0206
	Case isolation treatment 0.0137	Rapid treatment on the spot	0.0046
		Timely transport of the patient	0.0046
		When necessary, the isolation and resettlement area should be established, health protection and isolation should be conducted, and basic living supplies should be provided	0.0045
	Investigation of cases and suspected cases 0.0203	Determine the scope of the search and the object of investigation first	0.0030
		Determine if there are any protective measures before the investigation and whether the protective measures are in place	0.0030
		Determine whether the case questionnaire is comprehensive	0.0029
		Ask and fill in all items on the questionnaire	0.0028
		The results of the investigation were reported to the command department in time	0.0029
		Determine whether the survey is comprehensive and includes all respondents	0.0027
		Ensure that the content of the investigation was consistent with the elements of the incident	0.0029
	Case sampling 0.0794	Sample and suspected cases were sampled at the scene, and the preservation and transportation of the samples were also carried out	0.0082
		Determine whether personal protection is appropriate	0.0083
		Determine whether the sampling tool and the specimen delivery tool are reasonable	0.0080
		Sampling proficiency	0.0076
		Determine whether the types of samples are complete and whether the quantity is sufficient	0.0077
		Ensure that the test index is reasonable	0.0079
		Determine the timeliness and accuracy of field rapid diagnosis	0.0074
		After sampling, do sample records have to be filled out?	0.0081
		Delivery of infectious disease samples is in line with biosecurity requirements for Biosafety and handling of samples.	0.0083
		If necessary, when the command department discovers a positive result, does it report to the health administration department in time?	0.0078
	External environment survey 0.0580	Conduct hygienic investigation and sample water, food, etc.	0.0293
		Monitoring and harmless treatment of vectors and potentially contaminated media	0.0287
	Make use of local resources for rescue work 0.0427	When necessary, use local rescue forces (hospitals) and conduct social mobilization.	0.0427
	Event exploration 0.0203	Accuracy of the number of cases and the number of close contacts	0.0067
		Investigation of the source of infection	0.0067
		Identification of epidemiological causes	0.0069
	Field control of the epidemic situation 0.0263	Determine whether the case has been effectively treated.	0.0019
		Rational division of epidemic point and epidemic area	0.0019
		Proper control of the epidemic point	0.0019
		Proper management of the source of infection	0.0019
		Guiding and supervising hospitals to work well	0.0019
		Determine whether the contaminated area was sterilized	0.0019
		Determine whether disinfection is carried out as needed	0.0019
		Disinfection effect monitoring after disinfection	0.0018
		Recommendations for the management of high-risk groups	0.0019
		Is the management of the close contacts appropriate?	0.0018
		Was an emergency monitoring system established?	0.0018
		Determine whether the mode of transmission was effectively cut off.	0.0019
		Determine the need to carry out guidance for high-risk places (hospitals, etc.) and control suggestions for epidemic situation.	0.0019
		Provide health education for patients, close contacts and high-risk groups.	0.0018
	Preliminary report on the epidemic situation and judgment to the health administration department 0.0617	The report includes the following: preliminary judgment of events, preliminary control suggestions, problems and problems to be solved.	0.0617
	Public information release and media response 0.0137	Deal with the media correctly and communicate moderately in time.	0.0068
		Have a designated press spokesperson.	0.0069
	Emergency termination and aftermath 0.0137	According to the development of the incident and the implementation of prevention and control measures, when the termination condition of emergency response is reached, the emergency response shall be terminated, and the early warning shall be lifted.	0.0069
		Suggestions on terminating emergency response and releasing early warnings shall be made to the health administrative department when the emergency plans of foreign units are involved, and the termination conditions of emergency response are met.	0.0068
Summary report 0.1650	Internal communication 0.0094	Daily routine, daily log	0.0094
	Whether the content of the report is comprehensive 0.0296	It includes headlines, preface, event discovery and report, local natural/social factors, field investigation (case definition, epidemic intensity, epidemic characteristics, hygienic investigation), laboratory testing, investigation and analysis of risk factors, preliminary conclusions and basis of investigation, control measures and effect evaluation, existing problems and next work proposal.	0.0296
	Whether the content meets the requirements and whether the control measures proposed are scientific and pertinent 0.0205	Determine whether the content meets the requirements and whether the control measures proposed are scientific and pertinent.	0.0205
	Whether the conclusion is accurate 0.0554	Is the conclusion accurate?	0.0554
	Whether the report is in time 0.0373	Was the report delivered in a timely manner?	0.0373
	Internal reflection 0.0127	Carry out internal summarization in time and raise the deficiencies and rectification items of the unit’s emergency capability.	0.0127

The validity of the index system is as follows: (1) Content validity: According to the Delphi expert consultation method, there are eighty-six third-level indicators in the final index system. The average score of each index is 4.65 (4.18–5), the average CV is 0.12 (0–0.21), and the average percentage of full marks is 71.27% (41.18–100.00%) (Table 1). This shows that the content validity is good. (2) Index system reliability: In the total index system, the alpha reliability coefficient of the eighty-six indexes is 0.989>0.8, and the reliability is high. The alpha reliability coefficients of the internal indexes of the four major links are 0.939, 0.952, 0.988 and 0.902, indicating that the consistency of the indexes of the no-notice drill is good.

### Case analysis

To compare the implementation of some public health emergency drills in China, eight public health emergency comprehensive drills A1-A8 (see Table 4) with published papers and public data were selected, and the ranking of A1-A8 was calculated based on the new evaluation system constructed by the Delphi AHP in this study. The method is to score and rank the eight drills according to the weight C1-C4 of four primary indicators (see Table 5). Among them, C3 (field investigation and control) is listed as the most important index, with a weight of 0.3925.

**Table 4:** Overview of options.

options	data sources
A1	2015 cholera no-notice drill in Guangdong Province in 2015
A2	2016 plague no-notice drill in Foshan in 2016
A3	Emergency drill for school public health emergencies of a municipal health and family planning supervision institution
A4	Health emergency response team for prevention and control of sudden acute infectious diseases in Hubei Province
A5	Novel coronavirus pneumonia drill in a three grade a hospital
A6	Evaluation and analysis of emergency drill activities of disease control institutions in A county of Nanchang City
A7	Evaluation and analysis of emergency drill activities of disease control institutions in B county of Nanchang City
A8	Evaluation and analysis of emergency drill activities of disease control institutions in C county of Nanchang City

**Table 5:** Ranking of options.

first-level indexes name	weight	A1	A2	A3	A4	A5	A6	A7	A8
C1 Emergency preparedness	0.2775	90	92	95	70	70	100	87.2	84.1
C2 Verification and consultation of the epidemic	0.165	78.33	82	80	90	70	70	84	60
C3 Field investigation and control	0.3925	69.2	76.75	86	74	82	88	65	78
C4 Summary report	0.165	86.67	92	95	80	95	90	45	64.4
Total score		79.361	84.364375	88.9925	76.52	78.835	88.69	70.9955	74.47875
rank	R0	5	6	8	3	4	7	1	2

### Sensitivity analysis

In recent years, the sensitivity analysis of AHP has mostly been carried out through the change in the criterion weight coefficient, and the criterion selection generally only selects the index with the largest weight. In this study, "field investigation and control" has the greatest weight. The weight coefficient variation range of this criterion was 0.196–0.589, i.e., from -50% ~ 50%, with a 10% correction each time [38]; the value of this proportion change was allocated to other standards in proportion. This evaluation system was applied to score several public health emergency drills (A1-A8) with existing online public data and then determine the grade change of alternative schemes through the change in the "field investigation and control" index weight. The ranking of alternatives with different weight values is shown in Table 6.

**Table 6:** Sensitivity analysis results under different weights.

option	-50%	-40%	-30%	-20%	-10%	0	10%	20%	30%	40%	50%
A1	5	5	5	5	5	5	5	5	5	5	5
A2	7	7	6	6	6	6	6	6	6	6	6
A3	8	8	8	8	8	8	8	8	8	7	7
A4	4	4	4	4	4	3	4	3	3	2	2
A5	3	3	3	3	3	4	2	2	1	1	1
A6	6	6	7	7	7	7	7	7	7	8	8
A7	2	2	2	2	2	1	3	4	4	4	4
A8	1	1	1	1	1	2	1	1	2	3	3

By analyzing the results under different scenarios, it can be seen that the ranking of schemes has changed. Table 6 shows that the scheme ranking changes greatly under scenarios 0~10%. Under the scenario of -50% ~ -10%, the change in scheme ranking is small. The theoretical analysis is statistically verified by using Spearman correlation coefficient grade analysis [39], where Di represents the difference of ranks in a given scenario, and N is the number of pairs of ranks. The values of the Spearman correlation coefficient are given in the Table 7, and the results of "field investigation and control" under different weights are compared.

$$r=1-\frac{6\sum_{i=1}^{n}d^{2}}{n(n^{2}-1)}$$

**Table 7:** Value of Spearman correlation coefficient.

**weight**	**-50%**	**-40%**	**-30%**	**-20%**	**-10%**	**0**	**10%**	**20%**	**30%**	**40%**	**50%**
-50%	1	1	0.988	0.988	0.988	0.964	0.976	0.952	0.927	0.867	0.867
-40%		1	0.988	0.988	0.988	0.964	0.976	0.952	0.927	0.867	0.867
-30%			1	1	1	0.976	0.988	0.964	0.939	0.891	0.891
-20%				1	1	0.976	0.988	0.964	0.939	0.891	0.891
-10%					1	0.976	0.988	0.964	0.939	0.891	0.891
0						1	0.939	0.915	0.891	0.867	0.867
10%							1	0.988	0.976	0.927	0.927
20%								1	0.988	0.952	0.952
30%									1	0.976	0.976
40%										1	1
50%											1

It can be seen from the table that the Spearman coefficient is 0.867,1, and the correlation degree is very high. It shows that the developed model has little effect on the final ranking under the change of weight coefficient, so it has good applicability.

## Discussion

This study constructs n index system and determines the weight of factors through the Delphi and AHP, focuses on assessing the epidemic situation research, judging the situation and determining the management capacity of disease control personnel, and it provides more technical links and fewer process links with which to assess emergency response drills.

In the first round of consultation, many experts put forward suggestions on the modification of indicators, including the specialization of terms and ways to more accurately determine the scope of conditions, which resulted in deleted and added indicators. According to China’s national conditions and the division of responsibilities among health department personnel, the function of epidemic situation release is in the health administrative department, not in disease control. Therefore, the "timely and active release of information to the public" by disease control officials was deleted. The CDC can only initiate plans for its own unit, so this part of the plan was modified.

Research shows that emergency preparedness, especially material preparation, is the key link between public health emergencies and emergency response. In the first round of consultation, more than two experts believed that the material preparation should be further subdivided, and the weight should be increased. Therefore, combined with practical applications, the materials are further divided into communication equipment, life support materials and professional materials.

In addition, in combination with the focus of work at this stage, the "daily meeting and daily log" in the handling of epidemic situations are necessary in such situations, so experts suggested adding biological samples to play a decisive role in the diagnosis of cases, as well as the discovery of atypical cases and asymptomatic infections. Therefore, experts also suggested increasing the requirements for biological transportation of infectious disease samples.

At present, it is easy to ignore the description of the AHP in most of the Delphi analytic hierarchy research [16]. As the AHP is an important step in determining the index weight and an important part of the research method, combined with the structure of the current AHP research article, this study makes a detailed supplementary description of the content of the AHP, including the introduction of the preface and method process and the sensitivity analysis of the result.

## Advantages and limitations of this study

Compared with the current no-notice drill mode evaluation system for public health emergencies, the research subjects are hospitals, and the research subjects of the evaluation system developed in this study are public health departments such as the CDC, which makes up for the gap in this regard. At present, the no-notice drill mode evaluation system for public health emergencies is basically constructed by the literature research method. This study adopts the Delphi AHP, which is more scientific. However, compared with most AHP studies in recent years, this study has more secondary indicators, resulting in too many pairwise comparisons in the judgment matrix, which may have some information bias.

## Conclusion

The purpose of this study is to construct a set of evaluation index system of no-notice drills for public health emergencies is very important to improving the ability of public health department personnel to study, judge and deal with disease outbreaks like the pandemic. This is the first evaluation system to specifically query epidemiological investigators in the drill and research of a no-notice drill for public health emergencies, which is of great practical value to the CDC and other public health departments. The index focuses on the assessment of epidemiological investigation thinking, which plays an important role in improving the investigation and elimination ability of unexplained infectious diseases to give full play to the effect of emergency drills.

In this study, the Delphi method is used to screen the indexes, and the weight of each index is determined by the AHP and percentage method. It is more scientific than the literature analysis method used in most of the current research on drill evaluation systems. Through Spearman rank correlation sensitivity analysis, it is found that under the change in weight coefficient, the change in scheme ranking is small, which further shows the stability of the results.

As the scope of public health emergencies is still large, in future research, we will modify and conduct in-depth research on the more important epidemic situation of infectious diseases (such as COVID-19) in terms of the no-notice drill content of public health emergencies without to further improve the practicability and operability. When determining factor weights, several methods popular in the latest research have included the AHP, fuzzy evaluation method and the best-worst method; the calculation comparison is carried out at the same time to improve the scientificity and stability of the results.

## Supporting information

S1 File (ZIP)

S2 File (ZIP)
